# Secukinumab for the Treatment of Axial Spondyloarthritis: Long-Term Real-Life Data from Five Italian Referral Centers

**DOI:** 10.3390/jpm14111105

**Published:** 2024-11-14

**Authors:** Stefano Gentileschi, Carlo Cannistrà, Carla Gaggiano, Arianna Damiani, Linda Carli, Maurizio Benucci, Fabrizio Cantini, Laura Niccoli, Antonio Vitale, Caterina Baldi, Andrea Delle Sedie, Luca Cantarini, Marta Mosca, Bruno Frediani, Serena Guiducci

**Affiliations:** 1 Rheumatology Unit, Department of Medicine, Surgery and Neurosciences, University of Siena, 53100 Siena, Italy; 2 Department of Experimental and Clinical Medicine, Division of Rheumatology, University of Florence, 50134 Florence, Italy; 3 Rheumatology Unit, Department of Clinical and Experimental Medicine, University of Pisa, 56124 Pisa, Italy; 4 Rheumatology Unit, S. Giovanni Di Dio Firenze Hospital, 50134 Florence, Italy; 5 Rheumatology Unit, S. Stefano Hospital, 59100 Prato, Italy

**Keywords:** secukinumab, real-world evidence, axial-SpA, fibromyalgia

## Abstract

Background: This study aimed to evaluate the effectiveness and drug retention rate of secukinumab (SCK) in axial spondyloarthritis (ax-SpA) within a multicentric real-life cohort. Methods: Data from patients with ax-SpA treated with SCK at five Italian centers were collected retrospectively, excluding those with a diagnosis of Psoriatic Arthritis. Evaluations were conducted at baseline and at 3, 6, 12, 18, and 24 months. Assessments included C-reactive protein (CRP), erythrocyte sedimentation rate (ESR), BASDAI, and ASDAS-CRP. Results: Seventy-one ax-SpA patients (57.7% female, mean age: 53.86 ± 12.67 years) were enrolled. Baseline mean BASDAI was 6.2 ± 1.4 and ASDAS-CRP was 2.9 ± 1.3. Significant improvements in BASDAI and ASDAS-CRP were observed over time, with BASDAI reducing to 3.5 ± 1.9 (*p* < 0.0001) and ASDAS-CRP to 1.7 ± 0.9 (*p* < 0.0001) at 24 months. The follow-up duration averaged 20.46 ± 13.46 months. By the end of follow-up, 29.5% of patients discontinued SCK. The two-year retention rate was 72%. Dropout risk was higher in patients with fibromyalgia (HR: 2.896, *p* = 0.026). No significant retention differences were found based on sex, age, enthesitis, radiographic disease, combination with cDMARDs, SCK dosage, or previous bDMARD exposure. Lower ASDAS-CRP at the study’s end was noted in patients without fibromyalgia (1.4 vs. 2.5, *p* < 0.001). Conclusions: SCK showed rapid and lasting effectiveness for ax-SpA with a favorable retention rate, though fibromyalgia may reduce treatment persistence.

## 1. Introduction

Axial spondyloarthritis (ax-SpA) is a chronic inflammatory disease affecting the sacroiliac joint (SIJ) and the spine. The disease occurs in early adulthood, thus causing a long-standing disease and poor quality of life if not treated properly. Inflammatory back pain represents the hallmark of ax-SpA, but patients may also present extra-articular symptoms and comorbidities [1,2]. The classification of ax-SpA is based on the 2009 Assessment of Spondyloarthritis International Society (ASAS) criteria, which are used to differentiate between non-radiographic (nr)-ax-SpA and radiographic (r)-ax-SpA depending on the presence or absence of structural damage in the SIJ in the X-ray assessment [3]. The etiopathogenesis of the disease is still unclear. However, among other inflammatory mediators, the role of interleukin (IL)-17 is well known in the disease [4]. Secukinumab (SCK) is a human monoclonal antibody, approved for treating ax-SpA, which binds IL-17A, preventing the interaction between the interleukin and its receptor [5]. SCK had shown efficacy and safety in several phase 3 studies where it displayed an improvement in axial involvement with a long-term response from at least 52 weeks to 104 weeks [6,7]. SCK also presented significant efficacy and safety in several real-life studies [8,9,10] including data from an Italian register, confirming long-term clinical efficacy and long-term drug survival of SCK in patients with ax-SpA [11].

In this regard, we report our experience from a real-life cohort of patients affected by r-ax-SpA and nr-ax-Spa treated with SCK, focusing on the long-term drug retention rate and pointing out any potential differences in the drug survival due to clinical, therapeutic, or demographic characteristics.

## 2. Materials and Methods

Data from patients diagnosed with ax-SpA and treated with SCK from five Italian centers (Siena, Firenze Careggi, Firenze S. Giovanni Di Dio, Prato, Pisa) were retrospectively collected. All the enrolled patients were diagnosed with ax-SpA and classified according to the Assessment of Spondyloarthritis International Society (ASAS) criteria and patients were classified as nr-ax-SpA and r-ax-SpA according to pathological imaging findings on Magnetic Resonance Imaging or sacroiliac joint radiography.

Patients with a diagnosis of Psoriatic Arthritis (PsA) according to the Classification criteria for Psoriatic Arthritis (CASPAR) were excluded from the study. Both dosages of SCK (150 mg/4 w and 300/4 w) were employed, according to the clinician’s judgment. Laboratory and clinical data were retrieved from the patients’ charts corresponding to evaluations performed according to routine clinical practice at the following time points: baseline (T0), 3 months (T3), 6 months (T6), 12 months (T12), 18 months (T18), 24 months (T24), and at the last evaluation (LE) while on SCK treatment. Laboratory assessment included C-reactive protein (CRP) and erythrocyte sedimentation rate (ESR). Disease activity was assessed using the Bath Ankylosing Spondylitis Disease Activity Index (BASDAI) and the Ankylosing Spondylitis Disease Activity Score (ASDAS)-CRP. Active disease according to BASDAI was defined as >4. Disease activity according to ASDAS-CRP was defined as follows: 0 to 1.3 as remission, 1.3 to 2.1 as low disease activity, 2.1 to 3.5 as high disease activity, and >3.5 as very high activity. The presence of fibromyalgia was assessed according to the 2010 American College of Rheumatology Criteria [12].

The study’s primary aim was to evaluate SCK long-term drug survival in patients with ax-SpA. The secondary aims were as follows: to point out any different drug survival according to different clinical features, and to assess any alterations in drug survival correlated with the demographic and comorbidity characteristics of the patients or related to the number of previous lines of biologic therapy. The primary endpoint was evaluating the drug retention rate during the follow-up period. The secondary endpoints were to evaluate any difference in drug retention rate or clinimetric parameters in relation to the patients’ demographic characteristics, comorbidity, or previous lines of treatment.

Every enrolled patient signed informed consent, and the study received local IRB approval (CEAVSE protocol n° 19881, “SCK”). A statistical analysis was performed using the software R 3.5.2 GUI 1.70 El Capitan build. Continuous variables were represented by the mean and standard deviation after normality verification through the Kolmogorov–Smirnov test; categorical variables were described by a percentage and frequency distribution. The difference in clinometric indexes during the follow-up period (T3, T6, T12, T18, T24, last follow-up visit) was analyzed with a paired *t*-Test. The significance level was fixed at 5%. The survival analysis was performed through the Kaplan–Meier method. Bivariate and multivariate analyses were performed to assess the correlation between clinometric indexes (BASDAI and ASDAS-CRP) at the end of the follow-up and clinical variables.

## 3. Results

Seventy-one ax-SpA patients were enrolled (41 females, 57.7%, mean age ± SD: 53.86 ± 12.67 years). Baseline demographic, therapeutic, and clinical characteristics are summarized in Table 1. The demographic, clinical, and therapeutic data of our cohort, stratified by the presence or absence of fibromyalgia or r-axSpA, are available as supplementary material (Tables S1 and S2).

### 3.1. Clinical Response

**Figure 1:**
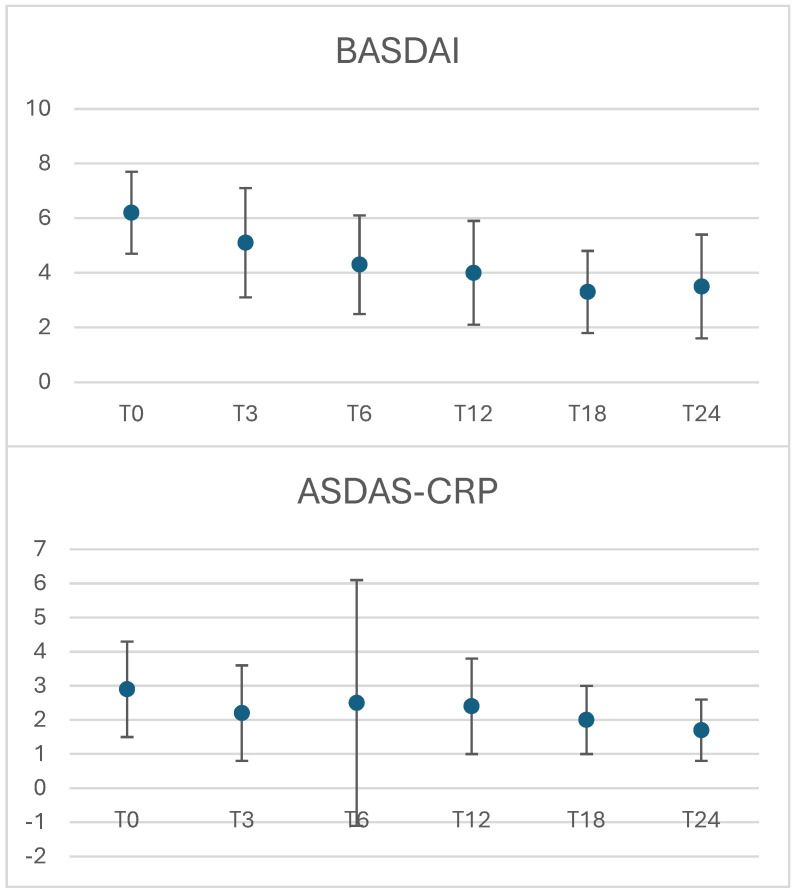
Changes in disease activity over time during secukinumab treatment: Bath Ankylosing Spondylitis Disease Activity Index (BASDAI), Ankylosing Spondylitis Disease Activity Score (ASDAS)-CRP.

The mean ± SD BASDAI at baseline was 6.2 ± 1.4, with 97.3% of patients presenting an active disease. The mean ± SD ASDAS-CRP at baseline was 2.9 ± 1.3 and 36.2% of patients had very high disease activity, 44.7% had high disease activity, 2.1% had low disease activity, and 15.0% were in remission. The mean ± SD BASDAI was 5.1 ± 2 (*p* < 0.001) at T3, 4.3 ± 1.8 (*p* < 0.001) at T6, 4 ± 1.9 (*p* < 0.001) at T12, 3.3 ± 1.5 (*p* < 0.001) at T18, and 3.5 ± 1.9 at T24 (*p* < 0.0001, all *p* values are intended when compared to T0). The mean ± SD ASDAS-CRP was 2.2 ± 1.4 (*p* = 0.04) at T3, 2.5 ± 3.6 (*p* = 0.63) at T6, 2.4 ± 1.4 (*p* = 0.11) at T12, 2 ± 1 (*p* = 0.01) at T18, and 1.7 ± 0.9 at T24 (*p* < 0.0001, all *p* values are intended when compared to T0). At T24, we found an upturn in patients reaching remission over the follow-up according to BASDAI evaluation (from 1/71 to 14/71 pts, *p* = 0.0005). At the same time, patients in remission and with low disease activity according to ASDAS-CRP increased up to 24 at the last follow-up visit compared with 9 patients at T0 (*p* = 0.01). CRP did not change significatively from baseline to T24 (0.89 ± 1 vs. 0.77 ± 1.2, *p* = 0.25), while the ESR significatively decreased (22.4 ± 20.1 vs. 16.5 ± 9.73, *p* = 0.03). Changes in disease activity over time are shown in Figure 1.

### 3.2. Safety

Concerning safety, two infectious adverse events were reported in our cohort. In detail, the two reported infection episodes were upper respiratory tract infections that did not require SCK to be discontinued. No *Candida* spp. infections were reported. Also, there were no occurrences of a uveitis onset or reactivation in our patients throughout the observation period. Similarly, no onset or reactivation of inflammatory bowel diseases were reported.

### 3.3. Retention Rate

In our cohort, the mean ± SD follow-up duration was 20.46 ± 13.46 months, ranging from 12 months to 60 months. At the end of follow-up, 21 patients (29.5%) suspended the treatment with SCK (6 for primary failure, 11 for secondary failure, 2 for adverse events, and 2 for other reasons).

**Figure 2:**
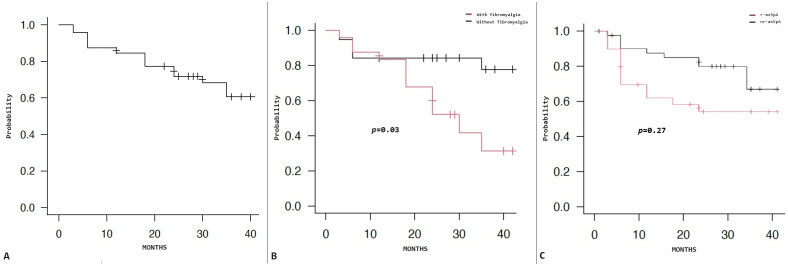
(**A**) Secukinumab cumulative survival. (**B**) Comparison of secukinumab survival between patients with and without fibromyalgia (*p* = 0.17). (**C**) Comparison of secukinumab survival between patients with radiographic (r) and non-radiographic (nr) axial spondyloarthritis (*p* = 0.27).

The two-year retention rate was 0.72 (95% CI: 0.59–0.82). Figure 2A shows the Kaplan–Meier survival curve of our cohort.

According to the survival analysis, the risk of dropout was significantly higher in patients with fibromyalgia, with a hazard ratio of 2.896 (95% CI: 1.136–7.385, *p* = 0.03, Figure 2B).

No differences in retention rate were observed dividing patients according to sex (*p* = 0.54), age range (<50 years old; 50–65; older than 65, *p* = 0.08), presence of enthesitis (*p* = 0.77), radiographic disease (*p* = 0.27, Figure 2C), combination treatment with cDMARDs (*p* = 0.89), SCK dosage (*p* = 0.12), or previous exposure to one or more other bDMARDs (*p* = 0.16).

### 3.4. Linear Regression

The bivariate analysis showed that ASDAS-CRP at the end of the study was significantly lower in nr-ax-SpA disease than in r-ax-SpA (1.4 versus 2.2, *p* = 0.02), and in patients without fibromyalgia (1.4 versus 2.5, *p* < 0.001). The multivariate analysis confirmed the correlation between higher values of ASDAS-CRP and fibromyalgia (*p* = 0.01). Conversely, the multivariate analysis did not confirm the association between higher ASDAS-CRP values and the presence of r-axSpA (*p* = 0.36).

## 4. Discussion

As of today, the use of biological therapies, such as tumor necrosis factor-alpha inhibitors and IL-17 inhibitors but also targeted synthetic DMARDs (i.e., Janus Kinase inhibitors), has revolutionized the treatment landscape for ax-SpA [13]. Specifically, SCK showed clinical efficacy and long-term drug survival both in registered controlled trials [6,7] and in a real-world context [9].

In this regard, this study aimed to provide additional real-life evidence on the therapeutic role of SCK in the treatment of ax-SpA, pointing out any differences in drug survival according to demographic, clinical, and therapeutic features. The decision to exclude individuals meeting the CASPAR criteria for PsA was prompted by recent increasing evidence indicating differing pathogenic characteristics between ax-SpA and axial PsA [14,15].

Concerning efficacy, in our cohort, both BASDAI and ASDAS-CRP values significantly improved during the follow-up, thus showing a prompt efficacy of SCK in significantly reducing BASDAI and ASDAS-CRP within the first 3 months of treatment, maintaining its efficacy through a long-term observation. Additionally, ASDAS-CRP diminished importantly at 18 months where more than 60% of patients had at least low disease activity. Similar data regarding clinical response rate and its subsequent maintenance at 2 years are reported in a recent multicenter Spanish study [16].

The SCK retention rate in our cohort at the two-year evaluation was 72%. These data are in line with other real-world experiences (Gentileschi et al., Ramonda et al., and Garcia-Dorta et al. with a 78,2%, 75%, and 70% two-year drug retention rate, respectively [8,9,17]) but also with data coming from multiple European ax-SpA registries [18] (two-year retention rate of 61%, Christiansen et al.).

Furthermore, it is interesting to note how the drug retention rate values that emerged from our cohort, as well as from other real-life studies, do not deviate too much from those reported in randomized trials, where study populations are selected based on restrictive criteria, particularly concerning comorbidities [5,6,7]. In this regard, a recent study from Danish rheumatological and dermatological registries showed how SCK had the second-best retention rate after Golimumab in patients with ax-SpA [19].

Concerning any differences in clinical response based on clinical or demographic features, in a recent study by the spondyloarthritis group of the Italian Society of Rheumatology, male sex was associated with a higher chance of BASDAI remission achievement, while female sex was associated with a higher chance of treatment discontinuation [9]. In the same study, patients with r-ax-SpA were more likely to achieve low disease activity according to both BASDAI and ASDAS. In opposition to that, within our cohort, we have not identified any significant variance in drug response or retention rate based on sex, while we found a reduced clinical response according to ASDAS-CRP in patients with r-ax-SpA. It is possible that the discrepancies observed between the two studies may be attributed to the smaller size of our sample, as well as our exclusion of patients who met the CASPAR criteria for the diagnosis of PsA.

It is known that the prevalence of fibromyalgia is higher among patients with rheumatic diseases compared to the general population [20]. This presents a significant obstacle when assessing disease activity in spondyloarthritis, impacting both patient-reported outcomes and clinical indexes [20]. Of note, in this study, fibromyalgia was a reason for reduced response according to ASDAS-CRP other than an augmented risk of drug discontinuation. These findings underscore the importance of providing comprehensive patient education, conducting more attentive follow-up, and adopting a holistic therapeutic approach for patients with non-inflammatory pain comorbidities. Such measures are essential for optimizing treatment adherence and reducing the risk of drug discontinuation.

This study has limitations, including a small sample size, a retrospective design, and the absence of a control group. Additionally, the cohort was skewed toward a difficult-to-treat phenotype, with most patients receiving SCK as a second or later line of biologic treatment. Specifically, our cohort included higher-than-expected proportions of female patients, older individuals, fibromyalgia cases, and HLA-B27-negative patients compared to typical ax-SpA epidemiology. These characteristics are indeed known to be associated with difficult-to-treat disease, TNFα inhibitor failure, and the consequent need for treatments with different mechanisms of action [21].

In conclusion, within our cohort, SCK demonstrated both rapid and sustained effectiveness in alleviating symptoms of ax-SpA with a robust persistence of the drug over time, regardless of age, sex, radiographic evidence of the disease, concomitant immunosuppressive therapies, or biologic line of treatment. On the other hand, our findings indicate that the presence of fibromyalgia could negatively affect the survival rate of SCK treatment, disclosing the need for measures aimed at optimizing treatment adherence in these patients.

## Figures and Tables

**Table 1:** Demographic, clinical, and therapeutic features of our cohort. List of abbreviations: r-ax-SpA, radiographic axial spondyloarthritis; cDMARDs, conventional disease-modifying anti-rheumatic drugs; bDMARDs, biologic disease-modifying anti-rheumatic drugs; HLA, human leukocyte antigen.

Demographic Features	Mean ± SD
Age (Years)	53.9 ± 12.7
Disease Duration	14.5 ± 10.0
Male/Female	30/41
**Clinical Features**	**N. patients (%)**
HLA-B27	27 (38)
Gut Involvement	1 (1.4)
Ocular Involvement	1 (1.4)
Peripheral Involvement	46 (64.8)
r-ax-SpA	29 (40.8)
Dactylitis	2 (2.9)
Enthesitis	36 (52.9)
Fibromyalgia	24 (33.8)
Hypercholesterolemia	11 (19.6)
Arterial Hypertension	15 (27.3)
Hyperuricemia	2 (3.6)
Hypertriglyceridemia	4 (7.1)
**Cardiovascular Comorbidities**	
▪ Angina ▪ Ischemic Disease ▪ Rhythm Disturbances ▪ Other	3 (5.4) 1 (1.8) 2 (3.6) 1 (1.8)
**Therapeutic Features**	**N (%)**
Previous cDMARD Exposure	40 (57.1)
Previous bDMARD Exposure	52 (75.4)
Concomitant cDMARDs	17 (23.9)
Secukinumab, 150 mg/4 w	53 (74.6)
Secukinumab, 300 mg/4 w	18 (25.3)

## Data Availability

All study data are available upon reasonable request to the corresponding author.

## Supplementary Materials

The following supporting information can be downloaded at: https://www.mdpi.com/article/10.3390/jpm14111105/s1. Table S1. Demographic, clinical and therapeutic characteristics of the cohort, stratified by the presence of fibromyalgia; Table S2. Demographic, clinical and therapeutic characteristics of the cohort, stratified by the presence of radiographic ax-SpA.
