# Supplementary material for: Secukinumab for the Treatment of Axial Spondyloarthritis: Long-Term Real-Life Data from Five Italian Referral Centers
Source: J Pers Med. 2024 Nov 14;14(11):1105. doi: 10.3390/jpm14111105 (PMC11595795; doi:10.3390/jpm14111105)
Supplement: Supplementary file 1 [file jpm-14-01105-s001.zip › jpm-3240541-supplementary.pdf]

Supplementary Material

Table S1. Demographic, clinical and therapeutic characteristics of the cohort, stratified by the presence of fibromyalgia.

	**Fibromyalgia**	**No fibromyalgia**
**Demographic Features**	**mean±SD**	**mean±SD**
Age (Years)	53.7±11.0	53.9±13.4
Disease Duration	10.1±5.6	17.1±11.2
Male/Female	9/15	19/19
**Clinical Features**	**N. patients (%)**	**N. patients (%)**
HLA-B27	8 (47.1)	17 (50.0)
Gut Involvement	0	1 (2.7)
Ocular Involvement	0	1 (2.7)
Peripheral involvement	15 (62.5)	26 (68.4)
r-ax-SpA	9 (37.5)	16 (42.1)
Dactylitis	1 (4.2)	0
Enthesitis	15 (62.5)	19 (52.8)
Hypercholesterolemia	2 (10.0)	9 (25.0)
Arterial Hypertension	4 (19.0)	11 (32.4)
Hyperuricemia	1 (5.0)	1 (2.8)
Hypertriglyceridemia	0	4 (11.1)
**Cardiovascular comorbidities**	**N (%)**	**N (%)**
▪ Angina	2 (10.0)	1 (2.8)
▪ Ischemic disease	0	1 (2.8)
▪ Rhythm disturbances	0	0
▪ Other	1 (5.0)	2 (5.6)
**Therapeutic Features**	**N (%)**	**N (%)**
Previous cDMARDs exposure	11 (45.8)	23 (60.5)
Previous bDMARDs exposure	21 (87.5)	25 (67.6)
Concomitant cDMARDs	7 (31.8)	9 (26.5)
Secukinumab 150 mg/4w	16 (66.7)	31 (81.6)
Secukinumab 300 mg/4w	8 (33.3)	7 (18.4)

Table S2. Demographic, clinical and therapeutic characteristics of the cohort, stratified by the presence of radiographic ax-SpA.

	r-ax-SpA	nr-ax-SpA
**Demographic Features**	**mean±SD**	**mean±SD**
Age (Years)	52.3±12.3	55.0±12.9
Disease Duration	16.4±10.0	12.8±9.9
Male/Female	8/21	22/20
**Clinical Features**	**N. patients (%)**	**N. patients (%)**
HLA-B27	9 (39.1)	18 (50.0)
Gut Involvement	0	1 (2.5)
Ocular Involvement	0	1 (2.5)
Peripheral involvement	18 (62.5)	28 (66.7)
Dactylitis	1 (3.6)	1 (2.5)
Enthesitis	16 (59.0)	20 (48.8)
Fibromyalgia	9 (36.0)	15 (40.0)
Hypercholesterolemia	5 (22.8)	6 (17.6)
Arterial Hypertension	7 (31.8)	8 (24.2)
Hyperuricemia	2 (9.1)	0
Hypertriglyceridemia	1 (4.5)	3 (8.8)
**Cardiovascular comorbidities**		
▪ Angina	1 (4.3)	2 (6.1)
▪ Ischemic disease	0	1 (3.0)
▪ Rhythm disturbances	0	0
▪ Other	2 (8.7)	1 (3.0)
**Therapeutic Features**	**N (%)**	**N (%)**
Previous cDMARDs exposure	15 (51.7)	25 (61.0)
Previous bDMARDs exposure	25 (86.2)	27 (67.5)
Concomitant cDMARDs	6 (21.4)	11 (29.7)
Secukinumab 150 mg/4w	21 (72.4)	32 (76.2)
Secukinumab 300 mg/4w	8 (27.6)	10 (23.8)
